# Evaluating the Verbal Behavior Milestones Assessment and Placement Program (VB-MAPP) Scores Using Principal Components Analysis

**DOI:** 10.7759/cureus.66602

**Published:** 2024-08-10

**Authors:** Tami Peterson, Jessica Dodson, Robert Sherwin, Frederick Strale

**Affiliations:** 1 Hyperbaric Oxygen Therapy, The Oxford Center, Brighton, USA; 2 Applied Behavior Analysis, The Oxford Center, Brighton, USA; 3 Hyperbaric Oxygen Therapy, Wayne State University School of Medicine, Detroit, USA; 4 Biostatistics, The Oxford Center, Brighton, USA

**Keywords:** cronbach’s alpha, vb-mapp, pca, applied behavior analysis (aba), internal consistency reliability, autism spectrum disorder (asd), verbal behavior milestones assessment and placement program, principal components analysis, autism and verbal behavior

## Abstract

Introduction

The Verbal Behavior Milestones Assessment and Placement Program (VB-MAPP) is an extensive tool used to assess children with autism and other developmental disabilities who have language delays. Applied behavior analysis (ABA) professionals frequently use the VB-MAPP to create personalized intervention programs catering to each child’s needs. The lack of studies examining the VB-MAPP at the pretest, posttest, and differential scores using principal components analysis (PCA) suggests an opportunity to conduct PCAs on these different VB-MAPP scores. In doing so, researchers could better understand the VB-MAPP's dimensionality and factor structure at these levels. This, in turn, could inform the development of more effective assessment strategies and intervention plans for individuals with language and social communication challenges.

Materials and methods

From January 2018 to July 2021, The Oxford Center in Brighton and Troy, Michigan, treated autistic children using ABA therapy. A convenience sample of 13 children was retrospectively analyzed using VB-MAPP, which evaluates various behavioral milestones using a pretest-posttest design. Descriptive data analysis and internal consistency reliability estimates (using Cronbach’s alpha) were calculated for pretest, posttest, and difference scores. A Wilcoxen signed-rank test was conducted to determine the statistical significance between the pretest and posttest. Correlation matrices were inspected for relevant relationships between VB-MAPP scales, and a PCA with orthogonal rotation was also performed on this pretest, posttest, and difference scores.

Results

The mean age for the children was 4.083 years ± 1.083 years, (95%CI 3.64, 4.36). Around 66.6% of the children had an autism severity level of three, 33.3% had a severity level of two, and none were at level one. Cronbach’s alpha for internal consistency reliability of the pretest, posttest, and difference scores, indicating excellent reliability with values of 0.948 for the pretest and 0.937 for the posttest, respectively. The difference scores had a lower but acceptable reliability coefficient of 0.752. PCA on the pretest scores identified three factors that explain 85.584% of the total variation, indicating that these components capture most of the data's structure. The posttest PCA also identified three factors, accounting for 84.293% of the variance, suggesting a similar complexity and good model fit as the pretest. PCA revealed four factors explaining 82.317% of the variation for the difference scores. The increase in factors suggests that changes between pretest and posttest scores are complex, likely due to the ABA treatment, and require an additional component to represent the data adequately. There is a good model fit; the underlying structure is more complex than the pretest or posttest alone.

Conclusions

Robust coefficient alphas combined with the shift to a more detailed factor structure post-ABA treatment highlight ABA therapy’s diverse and multi-faceted impact on children. The increase from three to four principal components indicates a richer and more nuanced pattern of improvements across different domains of verbal and social behavior. This detailed factor structure is a testament to the comprehensive and individualized nature of ABA treatment, reflecting the therapy’s effectiveness in addressing specific needs and fostering broad developmental gains in children.

## Introduction

Many assessment instruments have been developed and are currently in use to quantify and analyze the verbal behaviors exhibited by individuals diagnosed with autism spectrum disorder (ASD) [1]. These instruments have found widespread application in randomized controlled trials, ongoing registries, and observational studies, serving as reliable measures for evaluating verbal outcomes in ASD individuals [2]. 

In clinical settings, these tools play a pivotal role in characterizing the verbal operant associated with the symptoms and disabilities of autism [3]. They provide valuable insights into the challenges inherent in the disorder, thereby aiding in the diagnostic process [4]. Their utility extends beyond clinical environments. In the realm of research, these instruments have been, and continue to be, employed as outcome indicators. They are robust measures for tracking changes and improvements over time, providing researchers with quantifiable data to evaluate the efficacy of interventions and treatments. This dual functionality underscores the versatility and importance of these tools in both clinical and research contexts of ASD [5]. 

Many research efforts have been dedicated to examining the psychometric characteristics of a range of tools specifically developed to assess diverse behaviors in autistic individuals, more specifically, the measurement of verbal behaviors in individuals diagnosed with ASD. These studies, which determine speech, verbal, and language measurement scales, aim to ensure the reliability and validity of these instruments in capturing and quantifying the behavioral nuances associated with ASD. 

The Mullen Scales of Early Learning (MSEL) showed a median internal consistency reliability ranging from 0.75 to 0.83. The early learning composite had an internal consistency between 0.83 and 0.95. Test-retest reliability varied based on the age of the children [6]. The Autism Diagnostic Observation Schedule (ADOS) demonstrated median interrater reliability of 0.74-0.83 across four modules, with individual items ranging from 0.23 to 0.94. The total score interrater reliability was 0.85-0.92 [7,8]. The Assessment of Basic Language and Learning Skills-Revised (ABLLS-R) scores showed excellent inter-rater reliability, as Usry et al. [9] reported. 

The Autism Behavior Checklist (ABC) had high internal consistency, with Cronbach’s alpha ranging from 0.86 to 0.94. However, the inter-rater reliability was lower [10,11]. The Autism Diagnostic Interview-Revised (ADI-R) showed good internal consistency and high test-retest reliability. The inter-rater reliability was also high [12,13]. The Vineland Adaptive Behavior Scales (VABS) demonstrated high internal consistency reliability, with test-retest reliabilities mostly in the 0.80s to 0.90s. Inter-rater reliability varied [14,15]. 

The Autism Treatment Evaluation Checklist (ATEC) score and its four sub-scales showed high internal consistency. The Pearson split-half coefficient for the ATEC speech score was 0.942 [16]. The Childhood Autism Rating Scale (CARS) had good internal consistency with a Cronbach’s alpha of 0.94. After a 12-month interval, test-retest reliability was 0.88 for the total score [17-19]. The Simplified Chinese Psychoeducational Profile (C-PEP) showed high internal consistency reliability of the subtests and composites. The two-week test-retest reliability was 0.94 [20]. The Reynell Developmental Language Scale (RDLS) did not have publicly available internal consistency reliability estimates [21]. 

Reliability and validity studies are crucial for ensuring that a measure is consistent and accurately represents the construct it is intended to measure. These studies confirm that the scores obtained from a measure make sense based on the researchers’ understanding of the construct [22]. Once reliability and validity are established, researchers may move on to factor structure studies in the form of principal components analysis (PCA) to explore the dimensionality of the data. This involves identifying clusters of related items within a measure, which can provide insights into potential sub-constructs or dimensions [23]. 

PCA reduces the dimensionality of the data by transforming the original variables into a new set of uncorrelated variables called principal components. These components are ordered so that the first few retain most of the original variables' variability. This helps understand the data's underlying structure and interpret the relationships between variables [24]. Progressing from reliability and validity studies to factor structure studies allows researchers to understand their studied measures and the constructs better. It helps refine the measures, improve their utility, and provide deeper insights into the validity of the constructs of interest [25]. 

Given this, there is a noticeable lack of research investigations that employ PCA as a methodological tool for analyzing verbal behavior metrics in the evaluation of individuals diagnosed with autism. This suggests that the potential of PCA in this specific context may not have been fully explored or utilized in the field of autism research. 

PCA is a statistical procedure that could be useful in the study of autism, where it could help identify underlying patterns in the verbal behavior of autistic individuals. Despite the potential benefits of using PCA in this context, there appears to be a significant gap in the existing body of research. This could be due to various reasons, such as the complexity of the PCA method, the challenges associated with collecting and analyzing verbal behavior data from autistic individuals, or simply a lack of awareness about the potential applications of PCA in this field. 

Therefore, there is a clear need for more research studies that apply PCA to verbal behavior measures in the assessment of autistic individuals. This could lead to new insights and improved methods for evaluating and understanding autism, which could ultimately contribute to better diagnostic procedures, more effective interventions, and improved outcomes for individuals with autism. 

Rowsey et al. conducted a PCA on the Promoting the Emergence of Advanced Knowledge (PEAK)-Generalization module. Eighty-four children with autism were evaluated. The PCA yielded a four-factor solution that included the constructs of foundational learning and basic social skills, basic verbal comprehension, memory, and advanced social skills, advanced verbal comprehension, reading, and writing, basic problem-solving skills, and verbal reasoning, problem-solving, logic, and mathematical skills [26].

Researchers conducted a PCA on the ADI-R with 292 individuals with autism. A six-factor solution emerged: spoken language, social intent, compulsions, developmental milestones, savant skills, and sensory aversions. Five factors were significantly correlated with the validating measures and had good internal consistency, face validity, and discriminant and construct validity. Most intraclass correlations between siblings were adequate for use in genetic studies [27]. 

A study by Constantino et al. involved the analysis of autistic traits in a group of 226 child psychiatric patients, some of whom had pervasive developmental disorders. They used two main methods for their study: cluster analysis of data from the ADI-R and PCA of data from the Social Responsiveness Scale (SRS), a measure of autistic traits [28]. The findings suggested that there is a single, continuous factor underlying the various symptoms of autism, which can manifest differently across three main areas: social deficits, language deficits, and repetitive/stereotypic behaviors. The study did not find evidence to support the existence of separate subdomains of dysfunction within autism spectrum conditions [28]. 

VB-MAPP background

The Verbal Behavior and Milestones Assessment and Placement Program (VB-MAPP) synthesizes principles from applied behavior analysis (ABA), and B. F. Skinner’s theoretical framework offers a criterion-referenced assessment, curriculum guide, and skill-tracking system. This comprehensive tool is designed to evaluate and monitor the development of language and related skills in children with autism and other developmental disabilities. The VB-MAPP is structured into five distinct components: The Milestones Assessment measures 170 learning and language milestones across three developmental levels: 0-18 months, 18-30 months, and 30-48 months. It provides a detailed evaluation of a child’s progress in acquiring fundamental communication skills [29-31]. The Barriers Assessment identifies 24 common barriers that can impede learning and language acquisition. Practitioners can tailor interventions to address each child's specific challenges by pinpointing these obstacles. The Transition Assessment evaluates 18 areas to determine a child’s readiness to transition to a less restrictive educational environment. It helps plan smoother transitions and ensure the child is adequately prepared for new settings. The Task Analysis and Skills Tracking breaks down complex skills into smaller, teachable units and tracks the child’s progress over time. It allows for a granular approach to skill development, ensuring that each step is mastered before moving on to the next. The Placement and IEP Goals aid in developing individualized education program (IEP) goals. It ensures that the educational objectives are aligned with the child’s specific needs and abilities, facilitating targeted and effective intervention strategies [29-32].

PCA with VB-MAPP

Belisle et al. [29] ran a PCA on the VB-MAPP across 85 autistic participants. They reported that items did not yield factors consistent with the Skinnerian verbal operants, but rather, the items appeared to cluster in terms of skill complexity, producing a two-factor model.

The items appeared to cluster in terms of skill complexity, namely, Level 3 (30-48 months - Preschool), Level 2 (18-30 months - Toddlers), and Level 1 (0-18 months - Infants), producing a two-factor model. Their PCA indicated that the two components had eigenvalues greater than one; the components explained 74.168% and 10.694% of the total variance, respectively. Factor 1 variables and loadings consisted of Tact3 (1.033), Interverbal3 (1.033), LRFFC3 (1.029), Listener3 (1.025), Mand3 (1.011), Interverbal2 (0.898), Imitation2 (0.760), Tact2 (0.741), Mand2 (0.711), Reading3 (0.679), Listener2 (0.664), Echoic2 (0.641), and Writing3 (0.635). The remainder of Component 1 loadings were below 0.300 [29]. Factor 2 variables and loadings consisted of Tact1 (0.930), Listener1 (0.908), Echoic1 (0.863), Mand1 (0.789), Imitation1 (0.769), and LFRCC2 (0.405). The remainder of Component 2 loadings were below 0.400 [29].

With prior research showing untrained cross-operant transfers, results failed to support Skinner’s verbal behavior taxonomy distinguishing between the verbal operant categories as independent constructs, with implications for how behavior scientists and analysts describe language development and assess and treat language deficits of individuals with autism [29]. 

Skinner posits a six-factor verbal behavior model (his verbal behavior taxonomy): mands, tacts, echoics, intraverbals, autoclitics, and textuals. Belisle et al.'s PCA uncovered a two-factor model that aligned more with age-related entities, namely, Level 3 (30-48 months - Preschool), Level 2 (18-30 months - Toddlers), and Level 1 (0-18 months - Infants) skill levels [29,30].

Skinner’s verbal behavior taxonomy (operants) categorizes six different types of verbal behavior based on their functional relationships to antecedents and consequences as follows. Mands are requests or demands controlled by a need, reinforced by obtaining the requested item. Tacts are labels or descriptions of objects/events, evoked by their presence and reinforced by social acknowledgment [30]. Echoics are repetitions of what someone else has said, controlled by a verbal stimulus and reinforced by social approval. Intraverbals are responses to other verbal statements, controlled by prior verbal stimuli and reinforced by continued interaction. Autoclitics are modifiers that provide additional context or intent, such as “I think” to add uncertainty. Textuals are reading written words aloud, controlled by written stimuli and reinforced by social or educational outcomes [30].

The analysis of different VB-MAPP correlation matrices and the noted multicollinearity among VB-MAPP scales suggest that the categories of verbal operants might not be independent constructs. This finding contrasts with Skinner’s [30] assertions. Essentially, the verbal behaviors Skinner identified as distinct may be interrelated, as shown by the data from the VB-MAPP scale correlations. This could profoundly impact our comprehension and methodology of verbal behavior in behavior analysis. 

As chronicled above, exploratory research employing PCA to investigate the dimensionality of the VB-MAPP has been lacking. This includes an examination of its factor structure and an analysis of the internal consistency of its scales. The present study seeks to address this gap, with a primary objective to evaluate the factor structure of the VB-MAPP. This involves identifying common factors that are uncorrelated and distinct from each other, which would provide a clearer understanding of the underlying constructs measured by the VB-MAPP. 

In the existing body of literature, no known research has been conducted using PCA to analyze VB-MAPP scores in three distinct contexts: pretest scores, posttest scores with ABA intervention as the treatment, and difference scores, namely, the change in scores from pretest to posttest (effects of treatment). 

ABA is a scientific approach to understanding and modifying behavior that focuses on applying principles derived from behavioral science to improve socially significant behaviors. ABA's main objective is to increase positive behaviors and decrease negative behaviors, thereby enhancing the individual’s quality of life and ability to function independently. Methodologically, board-certified behavior analysts (BCBAs) and behavioral technicians assess and identify specific behaviors to target through detailed observation and data collection [31]. In terms of intervention, implementing strategies based on behavioral principles, such as positive reinforcement, to encourage desired behaviors. Therapists continuously monitor and measure behavior changes to evaluate the effectiveness of interventions. Therapists adjust and modify strategies as needed based on ongoing data analysis to ensure optimal outcomes. ABA is widely used, particularly in treating children with autism, to develop skills in areas like communication, social interaction, and daily living skills [31].

This gap in research is significant as PCA is a powerful statistical technique used to identify the underlying factors that explain the variance in a set of observed variables. In the context of VB-MAPP, conducting PCA on the pretest and posttest with ABA intervention as the treatment and difference scores (effects of treatment) could provide valuable insights into the structure and interrelationships of the verbal behavior milestones assessed by the tool. This, in turn, could inform the development of more effective assessment strategies and intervention plans for individuals with language and social communication challenges. 

## Materials and methods

Study location and subjects 

From January 2018 to July 2021, children diagnosed with autism were engaged, observed, and provided treatment at The Oxford Centers (TOCs), located in Brighton and Troy, Michigan, USA. TOCs are outpatient healthcare facilities that offer a wide array of clinical services tailored to address various conditions, including ASD. 

The range of services provided at TOC is extensive and includes ABA therapy, nutritional therapy, neurofeedback, music therapy, educational support, hyperbaric oxygen therapy (HBOT), and physical, occupational, and speech therapy. The individuals who receive treatment at TOC can benefit from any combination of these therapies. This approach allows for a personalized treatment plan that caters to each individual's unique needs. 

Data gathering 

A convenience sample was retrospectively gathered from electronic medical records by skilled research assistants, focusing on 13 children receiving ABA therapy. A pretest-posttest research design was utilized, with the initial pretest and post-test data from the VB-MAPP collected by three distinct BCBAs who were not authors of this study. All children involved in the study received ABA treatments. The study explored the records of children aged between two and six years. These records were scrutinized for potential inclusion in the study. The VB-MAPP verbal tests for both the pretest and posttest were administered to the children by a BCBA. Children diagnosed with a seizure disorder or any genetic or mitochondrial mutation were excluded from the cohort under study [31]. 

To safeguard the data’s validity, the investigators confirmed matched pairs to ensure congruence between each child’s pretest and posttest scores. This strategy validated that the child cohorts were identical except for the ABA intervention. This approach allowed the individual cohorts to serve as their own controls in the pretest-posttest comparison, thereby minimizing potential bias from extraneous variables [31]. 

ABA treatment

The experimental treatment in this research was ABA. This personalized therapeutic strategy aims to enhance the abilities of children diagnosed with ASD and empower them to flourish in their homes, schools, and communities. All children with autism were treated using a hybrid approach to ABA, which incorporated discrete trial training (DTT), mass trials, and naturalistic environment training treatment modalities [31-34].

DTT simplifies complexity by breaking down large tasks into small, individualized steps. It employs straightforward and systematic methods for teaching these tasks. Within DTT, mass trials involve repeatedly presenting the same stimulus until the learner responds correctly. Naturalistic environment training (NET), another form of ABA, teaches behavioral skills within a natural learning environment. It leverages the learner’s preferences and interests as motivation [31-34]. A blend of DTT, mass trials, and NET can significantly benefit autistic children by enhancing cognitive, language, social, and adaptive skill development. DTT helps autistic children learn appropriate responses to situations, improving communication and relationships with family, classmates, and peers. Skills like matching, discrimination, and imitation taught through DTT enhance learning that might be challenging to acquire in naturalistic settings. Mass trials expedite the acquisition of new behaviors by exposing autistic children to the same or similar stimuli repeatedly. This method strengthens memory and recall abilities, aiding in retaining learned behaviors over time. NET facilitates the transfer of generalization skills from DTT to different contexts (people, materials, and settings). Using naturally occurring reinforcements, NET enhances motivation, spontaneity, and engagement [31-34].

Before the training began, one of eight BCBAs designed a treatment plan for each child. These plans were customized to cater to each child's needs and goals, and each was assigned to one of 83 behavioral technicians. A team comprising three to five behavioral technicians was responsible for administering the ABA treatments to each child participant. Appropriate materials were selected and arranged in rooms where individual DTT and mass trials [31-34] were conducted or in a naturalistic setting where the child interacted with others and experienced functional and meaningful real-world situations. The assignment of behavioral technicians to child cohorts varied daily, with each technician delivering an average of four to seven hours of treatment per day. This ensured that each child received at least 25 hours of therapy per week. This approach underscores the commitment to providing comprehensive and personalized care to each participant in the study. 

VB-MAPP measure 

A BCBA administered the children with the VB-MAPP instrument at the pretest and posttest. There was a median of six months of ABA treatment administration time between the pretest and posttest, with a minimum of four months and a maximum of eight months. Each child was tested on behavioral milestone domains: mand, tact, listener, visual/perceptual skills, independent play, social, motor imitation, echoic, listener responding, intraverbal, group behavior, and linguistic structure. These skills are necessary for the acquisition of language and social skills. Each child was observed, prompted, and assigned a rating on a five-point Likert scale grid based on response behaviors recorded by the BCBA. The higher the score on the milestone sub-scale, the better the child's progress [35]. 

Each child was tested on various verbal milestone domains of the VB-MAPP, which are crucial for developing language and social skills. Each subject was observed, prompted, and assigned a pretest and posttest rating on a five-point Likert scale grid based on response behaviors recorded by the BCBA. A higher score on the milestone subscale indicates better progress for the individual cohort [35]. 

VB-MAPP milestone domains

Manding involves the speaker asking for what they want. For example, a teacher might ask a student, "What do you want?" and the student responds with "juice." In this scenario, the student has effectively requested a drink of juice. Developing manding skills is essential because it empowers the children to advocate for their wants and needs [35].

Tact refers to naming something. In the context of the VB-MAPP, tacts involve labeling or identifying objects, actions, or events in the environment. For instance, an individual points to a car and says, "car." An individual sees and verbally identifies a flower as a "flower." Developing tacts is crucial because they enable individuals to express their understanding of the world around them. It's like saying what they see, hear, or touch [35].

Listener responding (LR) assesses an individual's ability to respond to verbal stimuli from others. Essentially, it evaluates how well someone comprehends and reacts to spoken language. Listening is a critical skill for effective communication and social interactions. When a teacher says, "Point to the red ball," and the student correctly points to the red ball, the listener responds. Following instructions like "Give me the blue crayon" or "Show me the picture of a cat" also fall under this category [35].

Visual perceptual skills and matching-to-sample (VPMTS) assess an individual's ability to perform visual discrimination tasks. These tasks involve matching non-identical items, sorting by size, associating items, categorizing objects, completing patterns, and following sequential order. Visual perceptual skills are fundamental for understanding and interacting with the environment. Matching-to-sample tasks help individuals recognize similarities and differences between objects or pictures [35].

Independent play is an individual's ability to engage in play activities without direct interaction or guidance from others. It involves playing alone, exploring toys, and entertaining oneself. The importance is autonomy. Independent play fosters an individual's independence and self-sufficiency. It allows them to explore their environment and develop creativity and social skills. While independent, individuals learn to manage their emotions, solve problems, and entertain themselves. These skills contribute to their overall social development [35].

Social play assesses an individual's ability to play and interact with others. It focuses on social behaviors, cooperative play, and communication during shared activities. Social play is essential for developing social skills, understanding social cues, and building relationships. It involves turn-taking, joint attention, sharing, and understanding social rules [35].

Motor imitation refers to an individual's ability to imitate physical movements demonstrated by others. It involves copying actions, gestures, or motor patterns observed in the environment. Motor imitation is crucial for learning and social interaction. It allows individuals to learn by following and replicating movements made by peers, caregivers, or teachers [35].

Echoic assesses an individual's ability to repeat or echo auditory stimuli. It involves imitating spoken words or phrases after hearing them from someone else. Echoic behavior is a crucial building block for language development. It allows individuals to learn and practice verbal skills by imitating sounds and words [35].

Spontaneous vocalization refers to an individual's ability to produce verbal sounds or words without direct prompting or imitation. It involves spontaneously using language to express thoughts, feelings, or needs. Spontaneous vocalization is a critical milestone for language development. It allows individuals to communicate independently and share their experiences with others [35].

Listener responding by feature, function, and class (LRFFC) is an advanced type of listener-responding behavior that focuses on identifying objects based on specific characteristics. It involves recognizing objects by their associated features, functions, or categories (classes). When students are asked to "Touch something you eat" (function), they might touch food items. LRFFC helps children develop a deeper understanding of objects in their environment. It teaches them adjectives and verbs related to different objects [35].

Intraverbals refer to verbal behaviors where an individual responds to verbal stimuli from others without direct physical cues. Unlike echoing or repeating, intraverbals involve generating novel responses based on context. Examples are completing sentences (e.g., "Twinkle, twinkle, little ____") and answering questions (e.g., "What's your favorite color?") [35].

Group behavior assesses an individual's ability to engage in appropriate behaviors within a group or classroom setting. These behaviors include following group instructions, participating in group activities, and demonstrating social skills. Group behavior skills are essential for successful inclusion in educational and social environments. They allow individuals to interact effectively with peers, teachers, and classmates [35].

Linguistic structure assesses an individual's language complexity and grammatical skills. It focuses on various linguistic elements, including sentence structure, verb tenses, pronouns, and syntax. An individual saying, "I want juice" (using subject-verb-object structure) demonstrates the linguistic structure, and pronouns (e.g., "he," "she," "they") are correctly used in sentences [35].

Data analysis procedures 

The IBM SPSS Statistics for Windows, Version 29 (Released 2022; IBM Corp., Armonk, United States) was employed for all the descriptive and inferential statistics. All the subjects' demographic information and baseline (pretest) and posttest characteristics were collated and summarized. 

Summary statistics were generated for continuous variables, such as age and duration of ABA treatments. These statistics include the mean, standard deviation, median, and range. In addition, the number and percentage of subjects within each category were presented for all categorical variables, including race/ethnicity and autism severity. 

Internal consistency reliability estimates for the pretest, posttest, and difference scores with Cronbach's alpha were computed [36]. A hypothesis test on the pretest and posttest results was conducted using a Wilcoxen signed-rank test to verify the effectiveness of ABA. Alpha was set at .05, with statistical significance set at p<.05. Following this, a PCA was conducted on the pretest, posttest, and difference scores using the following steps. Data standardization involves scaling data, so each feature has a mean of zero and a standard deviation of one. A correlation matrix was computed to understand how each variable relates to each other [37]. A varimax rotation was implemented, an orthogonal rotation that maximizes the sum of the variance of the squared loadings (i.e., the correlation between the original variables and the component). This enhanced the clarity of the component interpretation.

Eigenvalue and eigenvector coefficients were computed from the correlation matrix to identify principal components. The eigenvalues were sorted in descending order and arranged for the corresponding eigenvectors. Eigenvalues greater than one were retained, resulting in a reduced-dimensional representation of the data [37]. This made subsequent analyses more manageable and interpretable. All the statistical results were reported comprehensively, using both text and table presentations for clarity and ease of understanding. 

Independent ethics committee

All participants in this study provided consent, either explicitly or through waiver. The research utilized data retrospectively collected from chart reviews conducted for clinical purposes. The study was reviewed by the WIRB-Copernicus Group (WCG IRB) and received an exemption (#1-1703366-1). The authors assert that this investigation poses minimal risk and adheres to the Belmont Report Regulations, specifically the 2018 Common Rule (45 CFR 46), Section 46 Subpart A, Basic HHS Policy for Protection of Human Research Subjects, 46.104 Exempt Research Paragraph d (1), (2), and (2) ii, and 46.117 Documentation of Informed Consent Paragraph c (1) (ii). Additionally, the study complies with the guidelines of the 1964 Declaration of Helsinki.

## Results

Demographics and paired samples tests

The mean age of the study participants was 4.083 ± 1.083 (95%CI 3.64, 4.36). About 66.6% of the children had an autism severity level of three, 33.3% had a severity level of two, and none were at level one. A considerable percentage of the sample subjects (70.0%) were classified as Caucasian, 9.0% were Hispanic, 6.0% were Middle Eastern, 3.0% were Native American, and 12.0% were unspecified. The intervention was delivered for those receiving ABA treatments with a mean of 5.833 months ± 0.835 months.

Internal consistency 

Internal consistency reliability coefficients in the form of Cronbach’s alpha for the pretest scores were r=0.948, n=13 items, which indicates excellent internal consistency reliability. For the post-test scores, r=0.937, n=13 items indicate excellent internal consistency reliability. For the difference scores, r=0.752, n=13 items indicate acceptable internal consistency reliability. 

ABA efficacy

As reported in Table 1, pretest-posttest statistical significance (p<.05) was obtained with the scales: mand, tact, LR, VPMTS, independent play, social play, motor imitation, spontaneous vocalization, intraverbal, group behavior, and linguistic structure. Non-significant differences (p>.05) were detected at echoic and listener responding by feature, function, and class (LRFCC).

**Table 1:** VB-MAPP scales descriptives and paired samples tests VB-MAPP: Verbal Behavior Milestones Assessment and Placement Program n=13 *Wilcoxen signed-rank test; **Effect size - Hedges' g Hedges' g criteria: ±0.20 = Low effect; ±0.50 = Moderate effect; ±0.80 = Large effect

VB-MAPP Scale	Mean ± SD	Median	p-value*	ES**	ES (95%CI)
VBMAPPMandPRE	2.958 ± 2.848	3.250	0.003	-1.542	-2.354,-0.703
VBMAPPMandPOST	4.333 ± 2.815	4.250			
VBMAPPTactPRE	3.000 ± 2.884	3.000	0.004	-1.032	-1.695,-0.341
VBMAPPTactPOST	4.167 ± 3.033	4.000			
VBMAPPLRPRE	3.917 ± 3.642	3.250	0.005	-1.185	-1.890,-0.453
VBMAPPLRPOST	5.708 ± 3.306	4.250			
VBMAPPVPMTSPRE	5.375 ± 3.491	4.250	0.007	-1.171	-1.872,-0.443
VBMAPPVPMTSPOST	6.7912 ± 3.115	5.500			
VBMAPPIndependentPlayPRE	5.625 ± 2.821	5.250	0.005	-0.905	-1.536,-0.246
VBMAPPIndependentPlayPOST	6.958 ± 2.562	7.000			
VBMAPPSocialPlayPRE	2.875 ± 2.144	2.250	0.003	-1.270	-1.999,-0.541
VBMAPPSocialPlayPOST	4.500 ± 2.286	4.000			
VBMAPPMotorImitationPRE	2.292 ± 2.210	1.500	0.007	-1.251	-1.974,-0.500
VBMAPPMotorImitationPOST	4.000 ± 2.440	3.250			
VBMAPPECHOICPRE	4.083 ± 4.252	3.000	0.059	-0.580	-1.147,0.009
VBMAPPECHOICPOST	4.292 ± 4.164	3.250			
VBMAPPSpontaneousVocalizationPRE	2.417 ± 1.743	2.250	0.011	-0.842	-1.460,-0.198
VBMAPPSpontaneousVocalizationPOST	3.125 ± 1.569	3.500			
VBMAPPLRFFCPRE	0.667 ± 2.015	0.000	0.109	-0.453	-1.003,0.114
VBMAPPLRFFCPOST	1.125 ± 2.068	0.000			
VBMAPPIntraverbalPRE	0.583 ± 1.294	0.000	0.011	-0.873	-1.497,-0.222
VBMAPPIntraverbalPOST	1.458 ± 1.658	1.000			
VBMAPPGroupBehaviorPRE	2.125 ± 2.247	1.750	0.007	-0.795	-1.402,-0.162
VBMAPPGroupBehaviorPOST	3.250 ± 1.752	3.500			
VBMAPPLinguisticStructurePRE	1.000 ± 1.706	0.000	0.027	-0.640	-1.218,-0.040
VBMAPPLinguisticStructurePOST	1.625 ± 2.432	0.500			

Effect sizes as measured by Hedges' g were large (>±0.80) for mand, tact, LR, VPMTS, independent play, social play, motor imitation, spontaneous vocalization, and intraverbal, with group behavior approaching a large effect at (-0.795). Hedges' g was moderate (±0.50) with echoic and LRFCC.

PCA on pretest scores 

The correlation matrix for the pretest scores had a correlation mean of r=0.621, a median of r=0.635, a minimum of r=0.047, and a maximum of r=0.970. The PCA on the pretest scores identified three principal components that collectively explain 85.584% of the total variation in the pretest data using the varimax rotated solution. At an eigenvalue cutoff point criterion of >1, these three factors capture most of the variability in the pretest scores, suggesting the data's underlying structure can be well-represented by these three components. A high explained variation of 85.584% indicates that most of the information in the pretest data is retained in the three factors, indicating a good model fit (Table 2).

**Table 2:** VB-MAPP pretest number of principal components VB-MAPP: Verbal Behavior Milestones Assessment and Placement Program n=13 Extraction method: Principal component analysis Rotation method: Varimax with Kaiser normalization Rotation converged in six iterations

Principal Component	Initial Eigenvalues	% of Variance	Cumulative %	Initial Extraction Sums of Squares Loadings Total	Cumulative %	Rotation Sums of Suares Loadings Total	% of Variance	Cumulative %
1	8.597	66.131	66.131	8.597	66.131	4.499	34.607	34.607
2	1.369	10.533	76.664	1.369	76.664	3.626	27.893	62.500
3	1.160	8.920	85.584	1.160	85.584	3.001	23.084	85.584
4	0.974	7.495	93.079					
5	0.263	2.021	95.101					
6	0.246	1.894	96.995					
7	0.197	1.513	98.508					
8	0.110	0.846	99.354					
9	0.064	0.495	99.848					
10	0.016	0.125	99.973					
11	0.003	0.027	100.000					
12	5.99E-17	4.61E-16	100.000					
13	-4.24E-16	-3.26E-15	100.000					

Principal Component 1 (PC1) is strongly associated with VBMAPP - ECHOIC - PRE (0.880), VBMAPP - Spontaneous Vocalization - Pre (0.870), VBMAPP - Linguistic Structure PRE (0.832), VBMAPP - Tact PRE (0.786), VBMAPP - Mand PRE (0.629), VBMAPP LR PRE (0.503), VBMAPP - Motor Imitation - PRE (0.576), and VBMAPP - Independent Play - PRE (0.579).

Principal Component 2 (PC2) is strongly associated with VBMAPP LRFFC - PRE (0.902), VBMAPP - Intraverbal - PRE (0.890), VBMAPP - Linguistic Structure PRE (0.424), VBMAPP - Social Play - PRE (0.691), VBMAPP LR PRE (0.675), and VBMAPP - Motor Imitation - PRE (0.607). 

Principal Component 3 (PC3) is strongly associated with VBMAPP - Group Behavior - PRE (0.907), VBMAPP VPMTS - PRE (0.814), VBMAPP - Independent Play - PRE (0.597), VBMAPP - Tact PRE (0.454), VBMAPP - Mand PRE (0.491), VBMAPP LR PRE (0.454), and VBMAPP - Motor Imitation - PRE (0.480). 

Table 3 shows factor loadings for the pretest rotated component matrix.

**Table 3:** Pretest rotated component matrix VBMAPP: Verbal Behavior Milestones Assessment and Placement Program; LRFFC: Listener responding by feature, function, and class; LR: Listener responding; VPMTS: Visual perceptual skills and matching-to-sample n=13 Extraction method: Principal component analysis Rotation method: Varimax with Kaiser normalization Rotation converged in six iterations

Variable	Principal Component 1	Principal Component 2	Principal Component 3
VBMAPP - ECHOIC - PRE	0.880		
VBMAPP - Spontaneous Vocalization - PRE	0.870		
VBMAPP - Linguistic Structure PRE	0.832	0.424	
VBMAPP - Tact PRE	0.786		0.454
VBMAPP - Mand PRE	0.629		0.491
VBMAPP LRFFC - PRE		0.902	
VBMAPP - Intraverbal - PRE		0.890	
VBMAPP - Social Play - PRE		0.691	
VBMAPP LR PRE	0.503	0.675	0.454
VBMAPP - Motor Imitation - PRE	0.576	0.607	0.480
VBMAPP - Group Behavior - PRE			0.907
VBMAPP VPMTS - PRE			0.814
VBMAPP - Independent Play - PRE	0.579		0.597

PCA on posttest scores 

The correlation matrix for the posttest scores had a correlation mean of r=0.540, a median of r=0.600, a minimum of r=-0.219, and a maximum of r=0.871. The PCA applied to posttest scores revealed, like the pretest, the presence of three main components, or factors. These factors collectively account for 84.293% of the total variation observed in the posttest data. When we consider an eigenvalue cutoff criterion greater than one, it becomes evident that these three factors encapsulate the majority of the variability present in the pretest scores. This suggests that these three components can adequately represent the data's inherent structure. The high explained variance of 84.293% indicates that a significant portion of the information from the pretest data is preserved within these three factors, signaling a good fit for the model. Although the explained variance is slightly lower than in the pretest PCA, it still indicates a strong representation of the data with three components. This suggests that the posttest data's structure is similarly complex to that of the pretest data (Table 4).

**Table 4:** VB-MAPP posttest number of principal components VB-MAPP: Verbal Behavior Milestones Assessment and Placement Program n=13 Extraction method: Principal component analysis Rotation method: Varimax with Kaiser normalization Rotation converged in 10 iterations

Principal Component	Initial Eigenvalues Total	% of Variance	Cumulative %	Extraction Sums of Squared Loadings Total	Cumulative %	Rotation Sums of Squared Loadings Total	% of Variance	Cumulative %
1	7.779	59.837	59.837	7.779	59.837	4.154	31.956	31.956
2	2.077	15.98	75.817	2.077	75.817	3.791	29.165	61.121
3	1.102	8.476	84.293	1.102	84.293	3.012	23.172	84.293
4	0.777	5.976	90.269					
5	0.410	3.157	93.427					
6	0.370	2.846	96.273					
7	0.259	1.996	98.269					
8	0.141	1.086	99.354					
9	0.062	0.476	99.831					
10	0.015	0.117	99.948					
11	0.007	0.052	100.000					
12	1.21E-16	9.32E-16	100.000					
13	-4.35E-17	-3.35E-16	100.000					

Principal Component 1 (PC1) is strongly associated with VBMAPP - Social Play POST (0.959), VBMAPP - Linguistic Structure POST (0.825), VBMAPP - Intraverbal POST (0.67), VPMAPP - Independent Play - POST (0.660), VBMAPP - Spontaneous Vocalization - POST (0.630), VBMAPP - Motor Imitation - POST (0.551), VBMAPP - ECHOIC - POST (0.569), and VBMAPP LR POST (0.598). 

Principal Component 2 (PC2) is strongly associated with VBMAPP - Mand POST (0.900), VBMAPP Tact POST (0.818), VBMAPP - Linguistic Structure POST (0.477), VBMAPP - Intraverbal POST (0.574), VBMAPP - Spontaneous Vocalization - POST (0.602), VBMAPP - ECHOIC - POST (0.715), and VBMAPP - VPMTS - POST (0.713). 

Principal Component 3 (PC3) is strongly associated with VBMAPP - Group Behavior - POST (0.898), VBMAPP - LRFFC POST (0.749), VBMAPP - Motor Imitation - POST (0.522), and VBMAPP LR POST (0.693).

Table 5 shows the posttest rotated component matrix.

**Table 5:** Posttest rotated component matrix VBMAPP: Verbal Behavior Milestones Assessment and Placement Program; LRFFC: Listener responding by feature, function, and class; LR: Listener responding; VPMTS: Visual perceptual skills and matching-to-sample n=13 Extraction method: Principal component analysis Rotation method: Varimax with Kaiser normalization Rotation converged in 10 iterations

Variable	Component 1	Component 2	Component 3
VBMAPP - Social Play POST	0.959		
VBMAPP - Linguistic Structure POST	0.825	0.477	
VBMAPP - Intraverbal POST	0.670	0.574	
VPMAPP - Independent Play - POST	0.660		
VBMAPP - Spontaneous Vocalization - POST	0.630	0.602	
VBMAPP - Motor Imitation - POST	0.551		0.522
VBMAPP - Mand POST		0.900	
VBMAPP Tact POST	0.472	0.818	
VBMAPP - ECHOIC - POST	0.569	0.715	
VBMAPP - Group Behavior - POST			0.898
VBMAPP - LRFFC POST			0.749
VBMAPP - VPMTS - POST		0.625	0.713
VBMAPP LR POST	0.598		0.693

PCA on the difference scores

The correlation matrix for the difference scores had a correlation mean of r=0.192, a median of r=0.224, a minimum of r=-0.414, and a maximum of r=0.719. The PCA on the difference scores identified four principal components, which together explain 82.317% of the total variation in the difference scores. The increase in the number of factors (from three to four) compared to the pretest and posttest PCAs suggests that the changes or differences in scores between the pretest and posttest are more complex as the result of the ABA treatment and therefore may require an additional component to adequately capture the variability in the data. The high percentage of explained variation (82.317%) indicates a good model fit and that these four factors still capture the majority of the information in the difference scores, although the underlying structure is more complex than either the pretest or posttest PCAs alone (Table 6).

**Table 6:** VB-MAPP difference number of principal components VB-MAPP: Verbal Behavior Milestones Assessment and Placement Program n=13 Extraction method: Principal component analysis Rotation method: Varimax with Kaiser normalization Rotation converged in seven iterations

Principal Component	Initial Eigenvalues	Total	Cumulative %	Extraction Sums of Squared Loadings Total	Cumulative %	Rotation Sums of Squared Loadings Total	% of Variance	Cumulative %
1	4.215	32.424	32.424	4.215	32.424	3.767	28.977	28.977
2	2.893	22.256	54.68	2.893	54.68	2.488	19.135	48.112
3	2.331	17.934	72.614	2.331	72.614	2.223	17.102	65.213
4	1.257	9.667	82.281	1.257	82.281	2.219	17.068	82.281
5	0.740	5.696	87.977					
6	0.656	5.046	93.023					
7	0.520	4.003	97.026					
8	0.198	1.522	98.548					
9	0.134	1.029	99.577					
10	0.034	0.263	99.84					
11	0.021	0.160	100.000					
12	-3.60E-16	-2.77E-15	100.000					
13	-5.59E-16	-4.30E-15	100.000					

Principal Component 1 (PC1) is strongly associated with VBMAPP Independent Play DIFF (0.894), VBMAPP VPMTS DIFF (0.813), VBMAPP Motor Imitation DIFF (0.789), VBMAPP Spontaneous Vocalization DIFF (0.673), VBMAPP MAND DIFF (0.530), VBMAPP Tact DIFF (0.508), and VBMAPP Social Play DIFF (0.452).

Principal Component 2 (PC2) is strongly associated with VBMAPP LR DIFF (0.824), VBMAPP LRFFC DIFF (0.776), VBMAPP ECHOIC DIFF (0.760), VBMAPP MAND DIFF (0.551), and VBMAPP Spontaneous Vocalization DIFF (-0.404).

Principal Component 3 (PC3) is strongly associated with VBMAPP Tact DIFF (0.805), VBMAPP Intraverbal DIFF (0.713), VBMAPP ECHOIC DIFF (-0.454), and VBMAPP Group Behavior DIFF (-0.667).

Principal Component 4 (PC4) is strongly associated with VBMAPP Linguistic Structure DIFF (0.870), VBMAPP Social Play DIFF (0.827), VBMAPP Intraverbal DIFF (0.634), and VBMAPP MAND DIFF (0.510).

Table 7 shows the difference scores rotated component matrix.

**Table 7:** VB-MAPP difference rotated component matrix VB-MAPP: Verbal Behavior Milestones Assessment and Placement Program n=13 Extraction method: Principal component analysis Rotation method: Varimax with Kaiser normalization Rotation converged in seven iterations

Variable	Component 1	Component 2	Component 3	Component 4
VBMAPPIndependentPlayDIFF	0.894			
VBMAPPVPMTSDIFF	0.813			
VBMAPPMotorImitationDIFF	0.789			
VBMAPPSpontaneousVocalizationDIFF	0.673	-0.404		
VBMAPPLRDIFF		0.824		
VBMAPPLRFFCDIFF		0.776		
VBMAPPECHOICDIFF		0.760	-0.454	
VBMAPPMANDDIFF	0.530	0.551		0.510
VBMAPPTactDIFF	0.508		0.805	
VBMAPPIntraverbalDIFF			0.713	0.634
VBMAPPGroupBehaviorDIFF			-0.667	
VBMAPPLinguisticStructureDIFF				0.870
VBMAPPSocialPlayDIFF	0.452			0.827

## Discussion

Pretest PCA discussion

The PCA performed on pretest scores identified three principal components (PC1, PC2, and PC3). These components collectively explain 85.584% of the total variation in the pretest data, implying that these three components capture the most critical information in the pretest scores.

PC1 (Verbal and Vocal Communication Factor) contains high loadings on variables related to echoics, spontaneous vocalization, linguistic structure, tacting, mands, and other related skills suggesting that this component captures a core aspect of verbal behavior and communication abilities. In other words, this component measures how well someone can communicate verbally and captures core verbal behavior and vocal communication skills [38]. 

PC2 (Language Comprehension and Social Interaction Factor) contains high loadings on LRFFC, intraverbals, social play, and motor imitation suggesting that this component captures abilities related to understanding and using language in a social context. This component measures how well someone can understand language and interact socially and captures language comprehension and the ability to use language socially [38]. 

PC3 (Group and Independent Behavior Factor) contains high loadings on group behavior, visual perceptual/motor skills, and independent play suggesting that this component captures skills related to engaging in group activities, motor skills, and playing independently. This indicates that this component measures how well someone can behave in a group setting, their motor skills, and their ability to play independently [38].

Posttest PCA discussion

Like the pretest, the PCA on posttest scores identified three principal components (PC1, PC2, and PC3). These factors collectively accounted for 84.293% of the total variance observed in the posttest data, implying that these three components encapsulate most posttest scores.

PC1 (Comprehensive Verbal and Social Interaction Factor) contains high loadings on social play, linguistic structure, intraverbals, spontaneous vocalization, and independent play suggesting that this component captures a wide range of verbal communication and social interaction skills. In other words, this component measures how well someone can communicate verbally and interact socially [38].

PC2 (Specific Verbal Operant Factor) focuses on mands, tacts, and echoics. The high loadings on these variables indicate that this component captures the specifics of verbal behavior regarding requesting (mands) and labeling (tacts). This means that this component measures how well someone can request things (mands), label or name things (tacts), and mimic verbal stimuli (echoics) [38].

PC3 (Group and Motor-related Behavior Factor) contains high loadings on group behavior, LRFFC, motor imitation, and LR, suggesting this component captures skills related to group activities and motor imitation abilities. This indicates that this component measures how well someone can behave in a group setting, their ability to respond to verbal instructions that involve identifying objects by their features, functions, or classes (LRFFC), their motor imitation skills, and their ability to respond appropriately to verbal instructions (LR) [38].

Difference PCA discussion

This PCA factor structure on the difference scores contains four principal components, which indicates a more complex underlying structure post-treatment, looking at pretest minus posttest differences.

PC1 (Independent and Social Interaction Skills) represents a comprehensive improvement in independent play, visual perceptual/motor skills, motor imitation, and spontaneous vocalization. Including mand (requesting), tact (labeling), and social play, it suggests broad gains in independent and social activities. This indicates that ABA treatment has broadly enhanced independent functioning and social interaction skills [38]. 

PC2 (Receptive Language and Verbal Imitation) focuses on listening and responding by function, feature, and class (LR and LRFFC), as well as echoic behavior (verbal imitation) and manding (requesting). This indicates specific improvements in receptive language skills and verbal imitation, suggesting that ABA treatment has notably strengthened the child’s ability to understand and respond to verbal cues and to engage in imitative verbal behavior [38].

PC3 (Specific Verbal Operants) highlights improvements in tacts (labeling) and intraverbal behavior (conversation skills) but with a negative association with echoic (verbal imitation) and group behavior. This suggests targeted gains in specific verbal operants, such as labeling and conversational skills, potentially at the expense of more rote verbal imitation and group interaction abilities [38].

PC4 (Linguistic Complexity and Social Utility) represents improvements in the complexity of linguistic structures, social play, intraverbals (conversational skills), and manding (requesting). This suggests that ABA treatment has facilitated more complex and socially integrated verbal interactions, enhancing both the structural aspects of language and the social utility of verbal behavior [38].

The PCA has reduced the complexity of the posttest data into four main components that capture the majority of the information. These components represent different factors related to independent play, visual perceptual/motor skills, motor imitation, spontaneous vocalization, receptive language skills, verbal imitation, labeling, conversational skills, and the complexity of linguistic structures. This simplification can help understand the underlying patterns in the data [38].

Effects of ABA treatment and factor structure increase

The increase in principal components from three to four in the difference scores suggests that ABA treatment has introduced a more nuanced pattern of improvements across different areas of verbal behavior and social interaction. Several factors could be driving this increased complexity. The analysis of the effects of ABA treatment and the observed increase in principal components from three to four in difference scores can be understood through a comprehensive breakdown of several potential factors and their implications [38]. 

Effects of ABA Treatment

ABA treatment is a widely recognized intervention for children with developmental disorders, particularly autism. Its primary goal is to enhance various behaviors, including verbal behavior and social interaction. The treatment involves systematic approaches to teaching new skills and reducing problematic behaviors, often tailored to individual needs [38].

Factor Structure Increase

PCA is a statistical technique used to reduce the dimensionality of data by transforming it into a set of uncorrelated variables called principal components. These components capture the maximum variance in the data with the fewest number of components. An increase in principal components from three to four in the difference scores after ABA treatment suggests that more distinct improvement patterns were captured [38].

Detailed Factors Driving Increased Complexity

ABA treatment may have led to significant gains across multiple domains, resulting in a broader scope of improvements. Initially, gains might have been generalized, captured by fewer components. With continued treatment, these improvements become more distinct and specific, requiring additional components to represent the diverse areas of enhancement accurately. Namely, independent play consists of improvements in playing alone and demonstrating self-reliance and creativity. Social play: Interactive play with peers improves, indicating better social integration and cooperation. Linguistic complexity: Advanced use of language, including grammar and sentence structure, showing cognitive and communicative development [38].

Differential Impact

ABA treatment does not uniformly affect all verbal behavior and social skills areas. Some children may show more significant progress in specific areas, while others may excel in different ones. This variability leads to a need for more components to capture the distinct patterns of change. Verbal behavior: Improvements in various types of verbal behavior, such as receptive language (understanding) and expressive language (speaking). Social skills: Differential social responsiveness, engagement, and interaction quality gains [38].

Interindividual Variability

The individual differences in how children respond to ABA treatment contribute to a more complex factor structure. Each child's unique trajectory of improvement means that more components are necessary to encompass the variability in the data. Individual responses: Some children may improve significantly in social interactions, while others show more progress in language skills [38].

Improvement in Specific Skills

ABA is known for targeting specific skills through tailored interventions. The improvements in these targeted skills become distinct enough to require separate components. LR: The ability to respond to instructions and questions. LRFFC: Understanding and responding based on the properties and functions of objects. Tacts: Labeling objects, actions, and events. Intraverbals: Engaging in conversations and responding to questions with related answers [38].

Development of Higher-Order Skills

Posttreatment, children might develop more complex and higher-order skills that were not present initially. These advanced skills necessitate additional components to adequately represent the nuanced improvements. Higher-order skills could include advanced problem-solving, abstract thinking, and complex social interactions that indicate higher cognitive and social functioning [38].

The shift to a more detailed factor structure post-ABA treatment highlights the therapy’s diverse and multi-faceted impact on children. The increase from three to four principal components indicates a richer and more nuanced pattern of improvements across different domains of verbal and social behavior. This detailed factor structure is a testament to the comprehensive and individualized nature of ABA treatment, reflecting the therapy’s effectiveness in addressing specific needs and fostering broad developmental gains in children [38].

The increase in principal components from the pretest and posttest (three components each) to the difference scores (four components) suggests that the changes observed due to the ABA treatment are more complex than the static measurements captured at a single point in time. The additional component in the difference scores indicates that ABA treatment impacts multiple areas of development, leading to a more intricate pattern of improvement that cannot be entirely encapsulated by the same three components identified in the pretest and posttest data [38].

This richer factor structure underscores the multi-dimensional effects of ABA therapy. Each principal component represents a distinct aspect of the children's development. The three components in the pretest and posttest likely capture broad domains such as basic communication skills, social interaction abilities, and general behavioral improvements. However, the fourth component in the difference scores may represent more specific or emergent areas of development that become evident only when comparing pre and posttreatment data. This could include finer aspects of verbal communication, nuanced social behaviors, or specific adaptive skills that develop from targeted interventions during ABA therapy [38].

Furthermore, the high explained variance (82.317%) by the four components in the difference scores suggests that despite the increased complexity, the model still fits well and captures most of the data's variability. This implies that ABA treatment is not only effective in producing broad improvements but also in fostering detailed and specific developmental gains that are critical for children's holistic development [38].

Strengths and implications

A significant strength of this study lies in its robust methodology. The comprehensive data collection involved using VB-MAPP scores from 13 children diagnosed with autism who received ABA therapy. Utilizing electronic medical records ensured the accuracy and reliability of the data. The study’s statistical rigor is evident through the application of PCA and the calculation of Cronbach’s alpha, which assessed both the dimensionality and internal consistency reliability of the VB-MAPP scores. High-reliability coefficients (0.948 for pretest and 0.937 for posttest) underscore the instrument’s consistency.

The detailed factor analytic procedures using PCA identified three factors in both pretest and posttest scores, explaining over 84% of the variance. This finding indicates that the VB-MAPP effectively captures the complexity of language and social behaviors. The emergence of four factors in the differential scores suggests intricate changes post-ABA therapy, reflecting a multifaceted impact on the children’s development.

One noteworthy implication of this study is the enhanced understanding of the VB-MAPP’s factor structure, demonstrating that the instrument is not only reliable but also capable of capturing nuanced changes resulting from interventions like ABA therapy. This research could inform intervention strategies as the identification of a richer factor structure in the differential scores implies that ABA therapy results in significant, diverse improvements across various domains. This supports the need for personalized intervention plans that address multiple areas of development in children with autism.

Future research directions might include expanding the sample size to further validate these findings and explore the generalizability of the identified factor structures. Longitudinal studies could investigate the long-term effects of ABA therapy on VB-MAPP scores, providing deeper insights into the sustained impact of interventions. Comparative studies, where researchers compare VB-MAPP with other assessment tools, could help determine its relative strengths and weaknesses, guiding more effective assessment and intervention planning.

Limitations

Despite the promising PCA results and strong reliability indicators, this study has several limitations that warrant consideration. One of the primary limitations is the sample size, which, although adequate for the internal analyses conducted, may not be large enough to generalize the findings to a broader population. Smaller sample sizes can inflate reliability coefficients and may not accurately represent the diversity of responses in a larger, more heterogeneous population. Future research should aim to replicate these findings with larger and more diverse samples to enhance the generalizability of the results.

While the internal consistency reliability of the pretest and posttest was excellent, the lower reliability of the difference scores (Cronbach’s alpha = 0.752) suggests some instability in the measurement of change. Difference scores are inherently more variable and susceptible to measurement error. This limitation highlights the need for caution in interpreting changes over time as the lower reliability could affect the robustness of the conclusions drawn regarding the treatment effects.

There are also limitations with factor analysis and model complexity. The PCA conducted for the pretest and posttest identified three factors each, accounting for a substantial portion of the variance (85.584% and 84.293%, respectively). However, the identification of four factors in the difference scores, explaining 82.317% of the variation, indicates an increased complexity in the data postintervention. While this may be attributed to the treatment effects, it also suggests that the postintervention data may be capturing additional underlying dimensions not present in the pretest or posttest alone. This complexity could complicate the interpretation of the factors and their relationships, necessitating further investigation into the nature of these additional components.

The study focuses on the effects of a specific ABA treatment, which may limit the external validity of the findings. The observed changes and the identified factors may be unique to this intervention and may not generalize to other treatments or settings. Future studies should compare different types of interventions to determine if the observed factor structures and reliability measures are consistent across various therapeutic approaches.

The study’s design, focusing on pretest and posttest measures, provides a snapshot of the treatment effects but does not account for the temporal stability of these effects. Longitudinal studies are needed to assess whether the changes observed are maintained over time and to explore the long-term impact of the intervention. Without longitudinal data, it is difficult to determine the persistence of the treatment effects and the stability of the identified factors.

The reliance on self-report measures for data collection may introduce subjective bias, affecting the reliability and validity of the results. Participants' responses could be influenced by social desirability, recall bias, or other subjective factors. Employing a mixed-methods approach, incorporating objective measures alongside self-reports, could mitigate these biases and provide a more comprehensive understanding of the treatment effects.

Finally, while PCA is a robust method for identifying underlying factor structures, it is sensitive to the number of items and the sample size. The interpretation of PCA results can be complex and may not fully capture the nuanced changes in the data. Other statistical techniques, such as confirmatory factor analysis (CFA) or structural equation modeling (SEM), could complement PCA and provide additional validation of the factor structures identified.

While this study provides valuable insights into the reliability and factor structures of pretest, posttest, and difference scores, the limitations outlined highlight the need for cautious interpretation and further research. Addressing these limitations in future studies will strengthen the findings and contribute to a more comprehensive understanding of the treatment effects and their implications.

## Conclusions

The excellent internal consistency reliability estimates for the pretest and posttest scores and the acceptable internal consistency reliability for the difference scores underscore the VB-MAPP's solid consistency, especially after the administration of the ABA treatments. The shift to a more detailed four-factor structure in the post-ABA treatment data (difference scores) reflects the ABA therapy's comprehensive and individualized approach. ABA is designed to address the unique needs of each child, and the nuanced improvements captured by the additional principal component underscore the therapy's ability to effect meaningful and multi-dimensional change. This finding reinforces the importance of personalized treatment plans in ABA therapy, which can lead to significant and varied developmental gains across multiple domains of behavior and functioning in children. By enhancing our understanding of VB-MAPP’s factor structure, this research contributes to optimizing assessment strategies and intervention planning, ultimately benefiting individuals with language and social communication challenges.
